# Precision Exercise in Type 2 Diabetes Mellitus: Targeting Signaling Networks for Lipid Homeostasis

**DOI:** 10.3390/metabo16040269

**Published:** 2026-04-16

**Authors:** Tan Tian, Feiyang Yu, Xingran Liu, Xuelin Zeng, Jianjun Yue, Shanjun Bao

**Affiliations:** 1 School of Sports Training, Wuhan Sports University, Wuhan 430079, China; 2024410706@whsu.edu.cn (T.T.); 2024410701@whsu.edu.cn (F.Y.); 2025410648@whsu.edu.cn (X.Z.); 2 School of Physical Education and Health, Guangxi Medical University, Nanning 530021, China; liuxingran@gxmu.edu.cn; 3 College of Physical Education, Anhui Normal University, Wuhu 241002, China; yuejj@mail.ahnu.edu.cn

**Keywords:** type 2 diabetes mellitus (T2DM), lipid metabolism disorders, exercise intervention, signaling pathway

## Abstract

Type 2 diabetes mellitus (T2DM) is frequently complicated by dyslipidemia, which accelerates insulin resistance and the progression of cardiovascular and hepatic diseases. While exercise intervention is a cornerstone of T2DM management, a systems-level understanding of its underlying molecular mechanisms remains incomplete. This article summarizes current evidence to propose that exercise functions as a signaling network regulator, concurrently modulating critical lipid metabolism-related signaling pathways: cyclic adenosine monophosphate (cAMP), phosphatidylinositol 3-kinase–protein kinase B (PI3K–AKT), forkhead box O (FOXO), and mitogen-activated protein kinase (MAPK) signaling pathways. We delineate how dysregulation of these signaling pathways contributes to lipid disorders in T2DM, highlighting their tissue-specific and often bidirectional roles. Subsequently, we detail the molecular adaptations induced by various exercise modalities—from aerobic training to high-intensity intervals—that restore homeostasis of this signaling network. By integrating these findings, we present a novel framework for precision exercise—defined as the tailoring of exercise modality, intensity, and volume based on an individual’s predominant signaling pathway disturbance, assessed via circulating or tissue-specific biomarkers. This framework advocates for future exercise prescriptions to be guided by molecular profiling alongside traditional physiological indicators. This mechanistic insight not only deepens our comprehension of exercise physiology but also paves the way for more effective, personalized strategies to combat T2DM and its metabolic complications.

## 1. Introduction

The global prevalence of diabetes presents a rapid increase, driven by factors such as population aging and dietary shifts. By 2050, the number of individuals with diabetes worldwide is projected to reach 1.31 billion [1]. Type 2 diabetes mellitus (T2DM), the predominant form, accounts for approximately 90% of all cases [2]. T2DM patients often exhibit pathological features of dyslipidemia, which are closely linked to insulin resistance [3]. When lipid metabolism is abnormal, excess fatty acids cannot be stored normally in adipose tissue, and accumulate in non-adipose organs such as skeletal muscle and liver, where they are converted into triglycerides and ceramides. This process inhibits downstream insulin signaling, ultimately leading to insulin resistance. Moreover, prolonged lipid metabolism disorders readily induce diabetic complications like non-alcoholic fatty liver disease (NAFLD) and coronary heart disease, posing serious threats to patient health [4,5]. Therefore, maintaining normal lipid metabolism is a crucial therapeutic strategy for T2DM and its complications.

Exercise intervention is a safe, cost-effective, and easily implementable foundational interventional strategy. Regular exercise at appropriate intensities has demonstrated favorable outcomes in the management of various conditions, including cardiovascular diseases, osteoporosis, and diabetes [6,7,8]. Exercise also optimizes lipid profiles by regulating triglycerides, total cholesterol, low-density lipoprotein (LDL) cholesterol, and high-density lipoprotein (HDL) cholesterol levels [9]. Additionally, it enhances cholesterol reverse transport capacity and reduces lipid deposition in the liver and skeletal muscle, thereby supporting vascular dilation and preserving endothelial function [10]. Thus, exercise serves as a crucial intervention approach for alleviating dyslipidemia and reversing the progression of T2DM. We delineate how dysregulation of these signaling pathways contributes to lipid disorders in T2DM, highlighting their tissue-specific and often bidirectional roles. Subsequently, we detail the molecular adaptations induced by various exercise modalities—from aerobic training to high-intensity intervals—that restore homeostasis within this signaling network. By mapping exercise-induced adaptations onto specific signaling networks, this review contributes to the emerging framework of “precision exercise,” which aims to tailor interventions based on individual molecular profiles.

## 2. Prediction and Screening of T2DM-Related Signaling Pathways

### 2.1. Acquisition of GEO Datasets

We accessed a dataset from the GEO database (https://www.ncbi.nlm.nih.gov/geo/) (GSE267853) containing gene expression profiles from T2DM patients with concomitant lipid metabolism disorders and healthy controls. This dataset includes three groups: healthy mice (8 weeks old), T2DM obese mice (8 weeks old), and T2DM obese mice treated with a vitamin D analog. Although human datasets for T2DM with dyslipidemia are limited, this mouse dataset was selected for its comprehensive representation of glucolipid metabolic disturbances, insulin resistance, and osteoporosis under T2DM pathological conditions, offering greater scientific rigor and data completeness when compared to other available datasets.

### 2.2. Plotting Volcano Plots and Heatmaps

**Figure 1:**
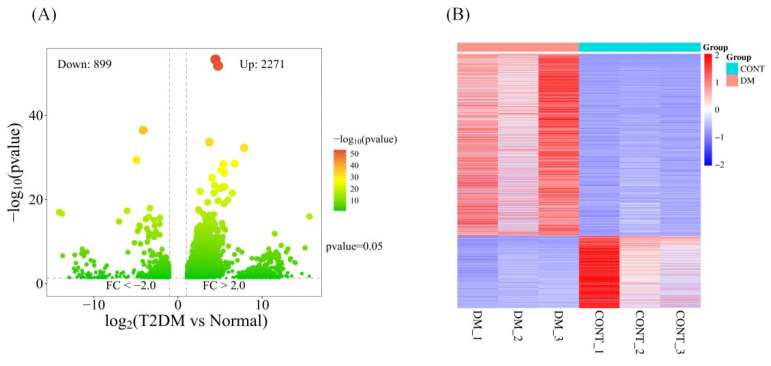
Volcano plot (**A**) and heat map (**B**) of differentially expressed genes.

The dataset was analyzed using the WebBioinformatics online platform (https://www.bioinformatics.com.cn/). A volcano plot and heatmap revealed that among 28,945 differentially expressed genes, 3170 genes exhibited significant expression differences, with 2271 upregulated and 899 downregulated genes (Figure 1).

### 2.3. GO Functional and KEGG Pathway Enrichment Analysis

**Figure 2:**
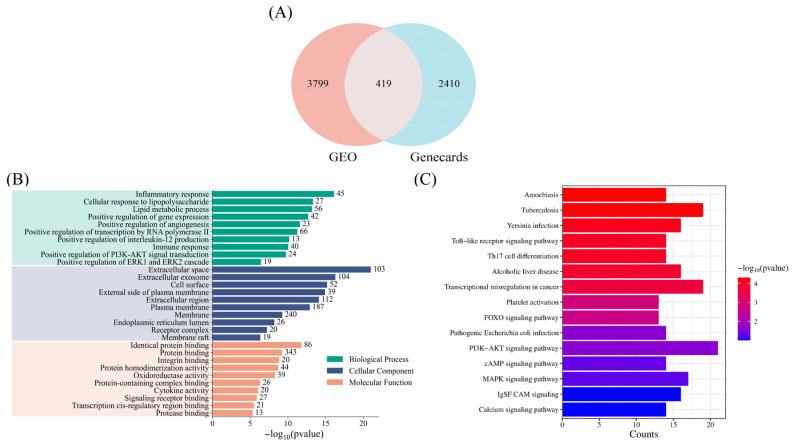
Differential expression genes analysis plots. (**A**) Venn diagram showing the intersection of differentially expressed genes from the GEO dataset (|logFC| > 0.5, *p* < 0.05) and predicted genes from the Genecards database, yielding 419 overlapping genes. (**B**) GO enrichment analysis (top 10 terms by *p*-value in BP, CC, and MF categories) showing significant enrichment of inflammatory response, immune response, extracellular space, and exosomes. (**C**) KEGG pathway enrichment analysis (*p* < 0.05, count > 12, followed by biological relevance filtering) identifying TLR, FOXO, PI3K-AKT, cAMP, and MAPK signaling pathways as significantly associated with T2DM-associated dyslipidemia. The TLR signaling pathway was excluded from further analysis due to limited literature on exercise regulation.

To further screen for potential signaling pathways associated with T2DM-related dyslipidemia and avoid overlooking relevant signaling pathways, we expanded the selection criteria. Genes with |logFC| > 0.5 and *p* < 0.05 in the GEO dataset were defined as significantly differentially expressed genes. These were then intersected with predicted genes from the Genecards database, yielding 419 overlapping genes, which were visualized in a Venn diagram (Figure 2A). Subsequent Gene Ontology (GO) and Kyoto Encyclopedia of Genes and Genomes (KEGG) enrichment analyses were performed using DAVID Bioinformatics (https://davidbioinformatics.nih.gov/home.jsp).

GO functional enrichment analysis was performed across three categories: biological process (BP), cellular component (CC), and molecular function (MF). The top 10 terms with the smallest *p*-values in each category were selected for presentation (Figure 2B). Significant enrichment was observed in terms such as inflammatory response, immune response, extracellular space, and exosomes, indicating that the differentially expressed genes are closely associated with inflammation, immune regulation, and cell signaling processes, and are largely localized to the cell membrane or extracellular environment. Notably, the PI3K-AKT and MAPK signaling pathways showed significant enrichment in this analysis.

For KEGG pathway enrichment analysis, two rounds of screening were applied. The first round used statistical thresholds of *p* < 0.05 and gene count > 12. The second round was based on biological relevance to T2DM-associated lipid metabolism disorders, with cross-referencing to the GO results. This process identified five significantly enriched signaling pathways: TLR, FOXO, PI3K-AKT, cAMP, and MAPK signaling pathways (Figure 2C). Among these, the TLR signaling pathway was not further analyzed, as current literature on its role in exercise-mediated regulation of T2DM-associated dyslipidemia remains limited. Therefore, this review focuses on the FOXO, PI3K-AKT, cAMP, and MAPK signaling pathways.

## 3. Lipid Metabolism-Related Signaling Pathways and the Maintenance of Lipid Homeostasis in T2DM

**Figure 3:**
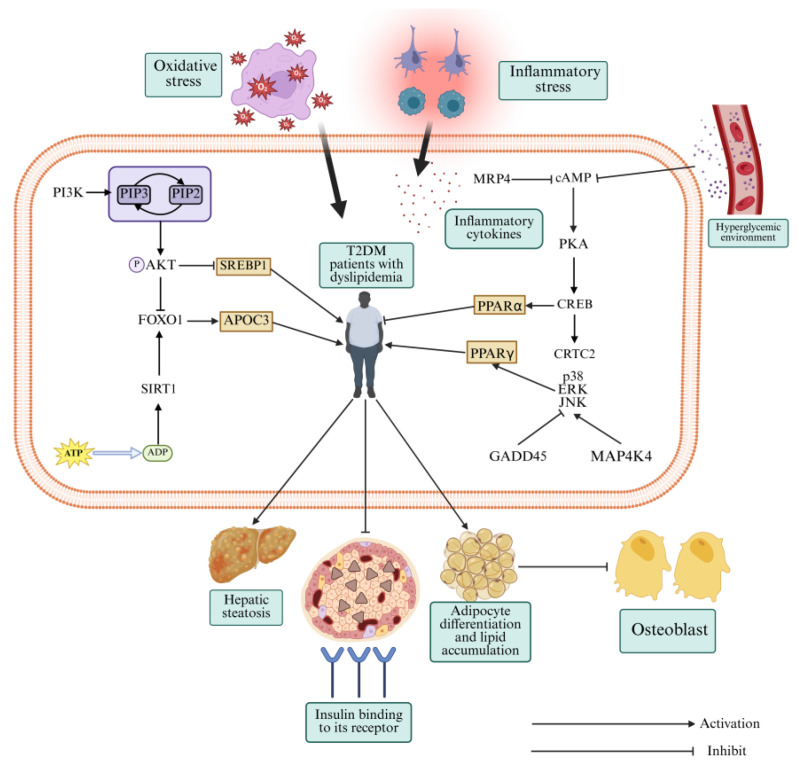
Regulatory mechanism of lipid metabolism-related signaling pathways in T2DM with dyslipidemia. cAMP, Cyclic adenosine monophosphate; CREB, cAMP response element-binding protein; JNK, c-Jun N-terminal kinase; PI3K, Phosphatidylinositol-3 kinase; PPARα, Peroxisome proliferator-activated receptor alpha; PPARγ, Peroxisome proliferator-activated receptor gamma; AKT, Protein Kinase B; SIRT1, Sirtuin1; FOXO, Forkhead box O; SREBP1, Sterol regulatory element-binding protein 1; APOC3, Apolipoprotein C3; MRP4, Multidrug resistance-associated protein 4; CRTC2, CREB-regulated transcription coactivator 2; ERK, Extracellular signal-regulated kinase; GADD45, Growth arrest-and DNA damage-inducible protein 45; MAP4K4, Mitogen-activated protein kinase 4.

Lipid homeostasis depends on the coordinated action of multiple signaling pathways. From our KEGG enrichment analysis, we focused on four signaling pathways including cAMP, FOXO, PI3K-AKT, and MAPK signaling pathways as core regulators of lipid metabolism in T2DM. MAPK, despite showing weaker enrichment than the other three, is a key downstream effector of insulin signaling. Below, we discuss how each of these pathways contributes to T2DM-associated dyslipidemia (Figure 3).

### 3.1. cAMP Signaling Pathway and Lipid Metabolism Disorders Associated with T2DM

cAMP is a ubiquitous second messenger [11] that transmits signals primarily by activating protein kinase A (PKA), which ultimately regulates the expression of downstream target genes [12]. The cAMP signaling pathway has been implicated in various diseases, including diabetes, Alzheimer’s disease, and NAFLD [13,14]. Therefore, maintaining normal cAMP signaling pathway is crucial for suppressing lipid metabolism disorders and treating T2DM.

Under physiological conditions, cAMP activates PKA, which phosphorylates cAMP response element-binding protein (CREB), thereby regulating downstream signaling. However, under pathological conditions such as glucolipotoxicity, oxidative stress, and inflammation, the cAMP signaling pathway is often dysregulated in T2DM patients [15]. Multidrug resistance-associated protein 4 (MRP4), an efflux transporter closely associated with glucose and lipid metabolism, plays a crucial role in diabetes and obesity [16]. MRP4 regulates lipid metabolism by suppressing cAMP signaling pathway. Following exogenous knockout of the *Mrp4* gene in mice, significant increases in the expression of cAMP, CREB, and CREB-regulated transcription coactivator 2 (CRTC2) expression were observed, along with dyslipidemia and adipose tissue inflammation [17]. This demonstrates that overactivation of cAMP signaling abnormally activates CRTC2, leading to increased lipid synthesis and inflammation, ultimately exacerbating lipid metabolism disorders and insulin resistance. Conversely, a previous study [18] reported that hyperglycemia and insulin resistance suppress cAMP expression in the liver, thereby impairing fatty acid oxidation and inducing hepatic steatosis. Elevating cAMP levels activates AMP-activated protein kinase (AMPK), which phosphorylates peroxisome proliferator-activated receptor alpha (PPARα), ultimately improving hepatic lipid metabolism disorders. Similarly, cAMP signaling is suppressed in the hippocampus and skeletal muscle of T2DM mice [19,20], and appropriate activators can enhance lipid metabolism and mitigate insulin resistance in these tissues. Therefore, cAMP signaling in T2DM exhibits unidirectional and tissue-specific regulation.

cAMP signaling in T2DM displays both suppression and overactivation depending on the tissue context. Both extremes exacerbate dyslipidemia. These observations suggest that restoring normal cAMP signaling by activation or inhibition depending on the tissue could help correct lipid metabolism in T2DM.

### 3.2. PI3K-AKT Signaling Pathway and Dyslipidemia in T2DM

The PI3K-AKT signaling pathway is a crucial downstream effector of insulin signaling, involved in cell growth, proliferation, differentiation, and apoptosis [21,22]. Normally, PI3K catalyzes the conversion of phosphatidylinositol-4,5-bisphosphate into phatidylinositol-3,4,5-trisphosphate [23], activating AKT phosphorylation, which enhances glucose and lipid metabolism and suppresses insulin resistance, ultimately contributing to the reversal of T2DM progression to some extent.

The PI3K-AKT signaling pathway regulates lipid metabolism by inhibiting lipolysis and promoting lipid storage, thereby enhancing adipose tissue fat-storing capacity [24]. This prevents excess fatty acids from accumulating in non-adipose tissues such as blood and liver, thereby reducing hyperlipidemia and insulin resistance [25]. In T2DM patients, impaired upstream insulin signaling typically suppresses the PI3K-AKT signaling pathway, contributing to lipotoxicity and increasing NAFLD risk [26]. The role of the PI3K-AKT signaling pathway in lipid homeostasis is closely linked to sterol regulatory element-binding protein 1 (SREBP1), a central regulator of lipid uptake and synthesis [27,28]. In high-fat diet (HFD)-induced T2DM rats, hepatic *SREBP1* mRNA levels were elevated, while insulin receptor substrate 2 (IRS2), PI3K, and AKT were reduced [29]. Papaya extract treatment also decreased hepatic SREBP1 expression, increased IRS2, PI3K, and AKT levels, and suppressed dyslipidemia. Similarly, gentianin improved hepatic lipid deposition and blood lipid profiles in T2DM mice by activating the PI3K-AKT signaling pathway and suppressing SREBP1 expression [30].

Thus, maintaining normal activation of the PI3K-AKT signaling pathway is a critical therapeutic target for lipid metabolism disorders associated with T2DM. Currently, this signaling pathway has been extensively studied in glucose metabolism [31]; its role in lipid homeostasis remains largely in the preclinical stage. Further clinical studies are needed to validate its therapeutic potential in humans. Additionally, when targeting this signaling pathway, attention may be paid to avoid excessive activation, which may promote tumorigenesis.

### 3.3. FOXO Signaling Pathway and Dyslipidemia in T2DM

FOXO proteins exist in four forms in mammals: FOXO1, FOXO3, FOXO4, and FOXO6 [32]. The FOXO signaling pathway is involved in cell survival, differentiation, apoptosis, and oxidative stress [33]. FOXO1 is primarily expressed in insulin-responsive tissues such as skeletal muscle, pancreas, and liver, playing a crucial regulatory role in lipid metabolism [34,35]. Thus, maintaining normal FOXO1 expression is essential for alleviating dyslipidemia and insulin resistance in T2DM.

The PI3K-AKT signaling pathway and the deacetylase enzyme Sirtuin 1 (SIRT1) are the primary regulators of FOXO signaling [36,37]. PI3K-AKT signaling activation phosphorylates and inhibits FOXO1, suppressing lipolysis and promoting lipid synthesis [38]. SIRT1, a key regulator of energy and lipid metabolism [39], is activated during exercise or energy restriction via AMPK-mediated increase in nicotinamide adenine dinucleotide levels [40]. SIRT1 deacetylates FOXO1, enhancing its nuclear localization and promoting lipolysis [41]. However, in T2DM, insulin resistance, oxidative stress, and glucolipotoxicity suppress PI3K-AKT signaling, thereby weakening AKT-mediated inhibition of FOXO1. This leads to FOXO1 overexpression, which activates apolipoprotein C-III transcription, thus stimulating triglyceride accumulation in blood and liver [42]. Moreover, SIRT1 expression is downregulated in obesity, as evidenced in HFD-induced obese mice, accompanying upregulated FOXO1 [43]. SIRT1 agonist administration restored normal FOXO1 expression, indicating the unidirectional FOXO1-mediated deacetylation. Similarly, reduced SIRT1 activity shifts the role of FOXO1 in exacerbated dyslipidemia from promoting lipolysis.

Overall, FOXO signaling exerts bidirectional regulation in lipid metabolism. Under normal conditions, AKT maintains FOXO at optimal levels, preventing excessive triglyceride deposition. Appropriate FOXO activation via exercise or other means can accelerate lipolysis and alleviate insulin resistance. Therefore, PI3K-AKT or SIRT1 agonists may be employed to modulate FOXO signaling toward a state beneficial for lipid homeostasis. Current research on this pathway primarily focuses on hepatic lipid metabolism. Consequently, future studies should expand to other tissues such as skeletal muscle.

### 3.4. MAPK Signaling Pathway and Dyslipidemia in T2DM

MAPK signaling pathway is another crucial regulator in lipid metabolism, comprising three branches: p38, c-Jun N-terminal kinase (JNK), and extracellular signal-regulated kinase (ERK) [44]. The p38/ERK signaling pathway primarily regulates lipid synthesis and metabolism, while JNK is closely associated with insulin signaling [45,46].

Mitogen-activated protein kinase 4 (MAP4K4) and growth arrest- and DNA damage-inducible protein 45 (GADD45) are key regulatory factors of MAPK signaling [47]. A study found that in high-sugar and HFD-induced T2DM mice, the expression of MAP4K4, p38, ERK1/2, and JNK was increased, while GADD45 expression was decreased in the liver. In contrast, artemether intervention normalized these protein levels and enhanced glucose and lipid metabolism [48]. On the other hand, GADD45 activation can promote p38 MAPK signaling in other diseases, thereby exacerbating disease progression [49], suggesting that the regulatory function of GADD45 is pleiotropic or tissue specific. Given that the mechanisms by which protein regulates MAPK signaling to maintain lipid homeostasis remains unclear, subsequent studies should be further explored in different tissues.

Peroxisome proliferator-activated receptor gamma (PPARγ), a transcription factor involved in adipocyte differentiation and lipid storage, is also regulated by MAPK [50]. One previous study reported increased mRNA expression of *PPARγ*, *ERK*, *p38*, and *JNK* in dyslipidemic mice [51]. Triterpenoid saponin treatment reduced the expression of these factors and restored dyslipodemia, suggesting that triterpenoid saponins may inhibit adipogenic differentiation by suppressing *PPARγ* expression via MAPK. However, MAPK can also inhibit PPARγ during osteoblast differentiation [52]. PPARγ also exhibits a close association with the PI3K-AKT signaling pathway [53], where its expression enhances PI3K-AKT activation, thereby promoting pancreatic β-cell survival and insulin sensitivity [54].

Taken together, the regulatory relationships among GADD45 and PPARγ, and MAPK signaling pathways exhibit highly context-dependent. Therefore, therapeutic targeting the MAPK signaling should be tissue- and disease stage-specific. For instance, PPARγ activators may be beneficial in the pancreas to enhance insulin signaling, while inhibitors may be preferred in adipose tissue to prevent excessive lipid accumulation.

## 4. Exercise Regulates Lipid Metabolism-Related Signaling Pathways to Alleviate Dyslipidemia in T2DM

**Figure 4:**
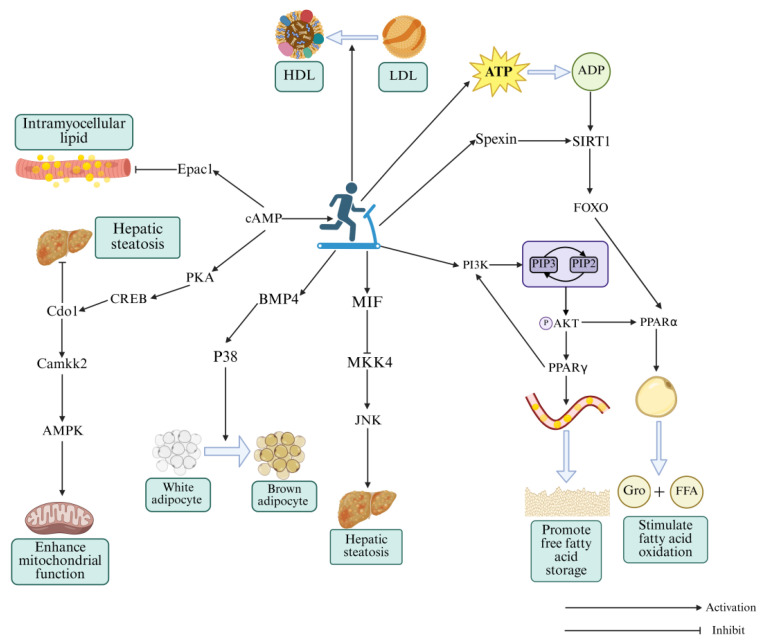
The molecular mechanisms underlying exercise-induced improvement of T2DM with dyslipidemia via regulation of lipid-metabolism related pathways. HDL, high-density lipoprotein; LDL, low-density lipoprotein; cAMP, Cyclic adenosine monophosphate; Epac1, Exchange protein activated directly by cAMP 1; PKA, Protein kinase A; CREB, cAMP response element-binding protein; Cdo1, Cysteine dioxygenase type 1; Camkk2, Ca^2+^/calmodulin-dependent protein kinase kinase 2; AMPK, AMP-activated protein kinase; BMP4, Bone morphogenetic protein 4; MIF, Macrophage migration inhibitory factor; MKK4, Mitogen-activated protein kinase kinase 4; JNK, c-Jun N-terminal kinase; PI3K, Phosphatidylinositol-3 kinase; PPARα, Peroxisome proliferator-activated receptor alpha; PPARγ, Peroxisome proliferator-activated receptor gamma; AKT, Protein Kinase B; SIRT1, Sirtuin1; FOXO, Forkhead box O.

Exercise is a cornerstone of clinical management [55]. Emerging “precision exercise” strategies aim to optimize its therapeutic potential by integrating multi-omics biomarkers and artificial intelligence for individualized risk assessment [56,57]. Unlike many pharmacological interventions, exercise acts through multiple signaling pathways simultaneously, with fewer organ-specific side effects. Although exercise is recognized as an important preventive and therapeutic measure for dyslipidemia in T2DM, its regulatory mechanisms during exercise interventions remain incompletely understood. Below, we summarize the molecular mechanisms by which exercise modulates the four signaling pathways discussed above, providing a theoretical foundation for future clinical research (Figure 4).

### 4.1. Exercise Activates cAMP Signaling Pathway for Restoring Dyslipidemia in T2DM

Abnormal cAMP signaling pathway in T2DM is a key contributor to dyslipidemia. Exercise can precisely regulate cAMP signaling pathway. The liver, a central regulatory organ for glucose and lipid metabolism, exhibits downregulated cAMP signaling pathway in T2DM, leading dyslipidemia and insulin resistance, which mutually reinforce each other and drive the progression of T2DM [58]. An 8-week aerobic exercise intervention improved hepatic lipid deposition and adipocyte degeneration in NAFLD mice by enhancing the cAMP-PKA-CREB signaling axis, promoting the expression of cysteine dioxygenase type 1 (Cdo1) [59]. Cdo1, a key enzyme in taurine synthesis, promotes energy expenditure and lipolysis, suppresses insulin resistance [60], and enhances AMPK signaling via interaction with Ca^2+^/calmodulin-dependent protein kinase kinase 2 (CaMKK2). Thus, exercise mitigates dyslipidemia through synergistic regulation of the cAMP-AMPK network. Long-term moderate-intensity exercise not only reduces hepatic lipid deposition and insulin resistance, but also enhances mitochondrial function, further promoting glucose and lipid metabolism, thereby reversing T2DM progression. Given the tissue-specific variability in the activation of the cAMP signaling pathway in T2DM patients, and the predominantly suppressed state of the AMPK signaling pathway under T2DM pathological conditions, it can be hypothesized that the cAMP signaling pathway bidirectionally regulates Cdo1 expression. However, whether both excessive and insufficient cAMP signaling suppress Cdo1 expression remains a hypothesis that requires direct experimental validation.

Beyond the cAMP-PKA signaling pathway, the exchange protein activated directly by cAMP 1 (Epac1) also serves as a downstream cAMP effector [61], regulating brown adipose tissue differentiation and energy expenditure [62]. Epac1 expression is suppressed in T2DM [63], but increased in skeletal muscle after moderate-intensity treadmill exercise [64], indicating that exercise enhances lipid oxidation via Epac1 activation. Given that both PKA and Epac1 are suppressed in T2DM skeletal muscle, they likely function synergistically rather than in isolation for maintaining lipid homeostasis.

Moderate-to-low intensity aerobic exercise can enhance lipid oxidation in tissues such as the liver and skeletal muscle by targeting the cAMP signaling pathway, thereby effectively attenuating T2DM-associated dyslipidemia. However, most current evidence is derived from preclinical rodent models, and human validation remains limited. Future studies should explore the effects of higher-intensity and resistance exercise on cAMP signaling, as well as the tissue-specific expression patterns of Cdo1 and Epac1 in patients with different T2DM phenotypes. Additionally, the hypothesized bidirectional regulation of Cdo1 by cAMP signaling requires direct experimental testing.

### 4.2. Exercise Upregulates PI3K-AKT Signaling Pathway for Attenuating Dyslipidemia in T2DM

Exercise interventions offer advantages over drug therapy in terms of reduced costs and fewer side effects. Aerobic exercise has long been regarded as a key intervention for obesity and insulin resistance [65]. Based on one of previous studies, 8-week aerobic exercise increased hepatic expression of PI3K, AKT and PPARγ in T2DM rats, which is beneficial to improve blood lipid levels, hepatic lipid deposition, and insulin sensitivity [66]. Thus, aerobic exercise activates the PI3K-AKT signaling pathway by PPARγ, enhancing adipocyte storage capacity, and creating a positive feedback loop.

High-intensity interval training (HIIT) also improves insulin sensitivity and HDL function [67]. An 8-week HIIT intervention upregulated PI3K, AKT, IRS-1, and PPARα in T2DM mice, while downregulated PPARγ to attenuate dyslipidemia [68]. These findings indicate that exercise interventions at varying intensities can activate the PI3K-AKT signaling pathway for the mitigation of dyslipidemia although PPARγ expression may vary depending on exercise intensity and disease stage. Notably, HIIT may exacerbate renal fibrosis in T2DM mice [69], highlighting the demand for personalized exercise prescriptions, especially in patients with complications such as diabetic nephropathy.

Collectively, the PI3K-AKT signaling pathway serves as a key target of exercise in regulating T2DM-associated dyslipidemia. Appropriate aerobic exercise should be a primary interventional approach for most T2DM patients, while HIIT should be prescribed with caution in those with complications (e.g., diabetic nephropathy). Notably, the expression of PPARγ in response to exercise shows inconsistent results across different exercise intensities and disease stages. Further studies are needed to clarify the mechanisms underlying these intensity-dependent effects and to determine whether PPARγ could serve as a predictive biomarker for personalized exercise prescription. Most of the current evidence remains preclinical so that clinical trials are urgently needed.

### 4.3. Exercise Enhances FOXO Signaling Pathway for Mitigating Dyslipidemia in T2DM

Currently, the studies on exercise-mediated FOXO regulation in dyslipidemia are relatively limited. Although FOXO reveals close links to PI3K-AKT and AMPK signaling pathways in T2DM [70,71], it still emerges as a promising therapeutic target during exercise intervention. SIRT1, a critical activator of FOXO, can be affected by intracellular energy changes [72], making exercise a potent modulator of SIRT1/FOXO signaling. According to a previous report [73], endurance exercise enhanced FOXO expression in Drosophila skeletal muscle, rescuing HFD-induced skeletal muscle damage. Even when FOXO expression is reduced, exercise suppresses dyslipidemia via SIRT1 activation, highlighting the redundancy of signaling pathways associated with lipid metabolism. HIIT also regulates FOXO signaling pathway via Spexin, an anorexigenic adipokine that promotes lipid metabolism and insulin sensitivity [74]. Similarly, an 8-week HIIT regimen increased Spexin, SIRT1, FOXO1, and PPARα in T2DM mice, mitigating dyslipidemia and insulin resistance [75]. Because Spexin can enhance SIRT1 expression [76], HIIT may activate the SIRT1/FOXO1 signaling pathway by boosted Spexin.

Together, the FOXO signaling pathway plays a crucial role in exercise-induced restoration of dyslipidemia in T2DM individuals. Aerobic exercise and HIIT have both been shown to activate the SIRT1/FOXO1 signaling axis via Spexin upregulation in preclinical rodent models. However, compared to other three signaling pathways, studies on the FOXO signaling pathway are relatively limited and remain at the preclinical stage. No human clinical trials have yet validated FOXO signaling pathway as a therapeutic target for exercise-mediated lipid regulation. Future studies should prioritize translational research to confirm whether FOXO activation in skeletal muscle and liver translates to improved lipid profiles in T2DM patients, and to determine the optimal exercise modality for FOXO modulation.

### 4.4. Exercise Triggers MAPK Signaling Pathway for Ameliorating Dyslipidemia in T2DM

MAPK signaling is an evolutionarily conserved pathway that controls cell proliferation, metabolism, and apoptosis [77,78]. Dysregulation of this pathway has been implicated in rheumatoid arthritis, cancer, and T2DM [79,80]. Although previous studies have demonstrated that exercise can reverse disease progression by inhibiting abnormal MAPK signaling pathways, systematic exploration in T2DM accompanied by concomitant lipid metabolism disorders remains limited. Therefore, the following section will summarize studies on exercise-induced activation of the MAPK signaling pathway for rescuing dyslipidemia in T2DM.

Macrophage migration inhibitory factor (MIF), a pro-inflammatory cytokine, can also modulate insulin signaling and lipid metabolism [81]. NAFLD is a chronic metabolic disorder sharing core pathological environments with T2DM, such as insulin resistance and lipid metabolism disorders [82]. A 16-week swimming training upregulated MIF expression in NAFLD mice through inhibiting mitogen-activated protein kinase kinase 4 (MKK4) and suppressing JNK phosphorylation, thereby ameliorating hepatic lipotoxicity [83]. However, MIF is often elevated in T2DM and exhibits context-dependent effects [84,85], warranting further investigation. Moreover, lipid metabolism disorders are frequently accompanied by the whitening and dysfunction of brown adipose tissue [86], a pathological state closely linked to dysregulated bone morphogenetic protein 4 (BMP4)/p38 signaling pathway. It was found that HFD induction can suppress BMP4 and p38 in mouse adipose tissue, but aerobic exercise can rescue the suppression of these signaling pathways [87], thereby stimulating the brown adipose tissue function and lipid metabolism. Interestingly, these findings are contradictory to the conventional view that suppressing excessive p38 activation is beneficial in T2DM, highlighting exercise’s core to counteract pathological stress with physiological activation. It is noteworthy that MIF and BMP4 are part of a broader class of exercise-induced signaling molecules, known as exerkines, which are increasingly recognized as key mediators of exercise benefits, potentially even regulating processes like ferroptosis [88]. Future research should explore whether the amelioration of T2DM-related dyslipidemia by exercise involves exerkine-mediated modulation of cell death and metabolic pathways. The molecular mechanisms described above are summarized to clearly illustrate critical regulatory roles of exercise in T2DM with dyslipidemia (Table 1).

Thus, unlike many drugs that broadly inhibit or activate a given signaling pathway, aerobic exercise appears to modulate MAPK signaling in a context- and tissue-specific way, which may be particularly suitable for treating dyslipidemia in T2DM. The divergent roles of p38, JNK, and ERK branches, as well as the context-dependent effects of MIF and BMP4, highlight the need for caution when designing exercise prescriptions targeting this signaling pathway. Future investigations are highly desired to explore the ERK signaling pathway in exercise-induced optimization of lipid metabolism, and to determine whether MAPK-related biomarkers (e.g., MIF, BMP4) can be reliably used to monitor exercise efficacy in clinical settings. Currently, all evidence remains at the preclinical stage.

## 5. Conclusions

The prevalence of T2DM is increasing rapidly worldwide, and dyslipidemia remains a core pathological feature driving insulin resistance and disease progression. Although novel pharmacotherapies such as mazdutide and lanifibranor have shown promise in clinical trials, their long-term safety remains to be established. Mainstream drugs such as metformin and semaglutide are effective but are often limited by gastrointestinal side effects, hypoglycemia, and variable efficacy. Moreover, when compared with oral pharmacotherapy, exercise compliance poses a major challenge. Therefore, a combinatorial strategy integrating pharmacotherapy with individualized exercise prescription is recommended for optimal T2DM management.

This article synthesizes current evidence demonstrating that exercise acts as a signaling network regulator, concurrently modulating the cAMP, PI3K-AKT, FOXO, and MAPK signaling pathways to restore lipid homeostasis in T2DM. A key contribution of this article is the identification of eight “exercise effector factors” (Cdo1, Epac1, PPARγ, PPARα, SIRT1, Spexin, BMP4, and MIF) that could serve as molecular readouts of signaling pathway-specific exercise adaptations.

Currently, exercise prescriptions for T2DM patients are primarily guided by traditional indicators such as blood glucose, heart rate, and maximal oxygen uptake (VO_2_max). While these indicators are cost-effective, easy to measure, and provide real-time feedback on exercise safety and intensity, they do not capture whether exercise has truly improved the underlying pathological state of dyslipidemia at the tissue or molecular level. In contrast, the proposed exercise effector factors (summarized in Supplementary Table S1), although more challenging to measure and not suitable for real-time monitoring, could complement traditional indicators by revealing the molecular efficacy of exercise interventions on specific signaling pathways and tissues.

Therefore, clinical translation and future directions may be summarized as follows:

For patients with predominant hepatic cAMP suppression (reflected by low Cdo1 expression), moderate-intensity aerobic exercise may be prioritized.

For those with reduced SIRT1-FOXO activity, HIIT could be considered if no contraindications (e.g., diabetic nephropathy) are present.

The expression levels of these effector factors could be measured at baseline and during follow-up to guide exercise prescription adjustments.

Most mechanistic evidence presented in this article is derived from preclinical animal models; human validation is urgently needed before these biomarkers can be integrated into clinical practice. Furthermore, given the tissue-specific and context-dependent nature of these signaling pathways in T2DM, future studies should clarify the inconsistent findings across different tissues and disease stages. By combining molecular biomarkers with traditional physiological indicators, we envision a future of precision exercise that delivers more effective, personalized, and scientifically grounded interventions for T2DM and its metabolic complications.

## Figures and Tables

**Table 1:** Molecular mechanism by which exercise alleviates T2DM with dyslipidemia through regulating lipid metabolism-related signaling pathways.

Number	Target Gene	Changing Pattern	Function
1	Cdo1	↑	Attenuating hepatic lipid accumulation and enhancing mitochondrial function.
2	Epac1	↑	Reducing lipid accumulation in skeletal muscle and triggering insulin sensitivity.
3	PPARγ	↑	Activating PI3K-AKT signaling pathway to promote lipid storage capacity in adipocytes.
4	PPARα	↑	Enhancing fatty acid oxidation and reducing lipid accumulation.
5	SIRT1	↑	Upregulating FOXO expression and promoting lipid utilization.
6	Spexin	↑	Stimulating lipid metabolism and inhibiting hepatic lipid accumulation.
7	BMP4	↑	Upregulate p38 and triggering browning of white adipose tissue.
8	MIF	↑	Inhibiting JNK signaling pathway to ameliorate hepatic steatosis.

Note: ↑, Upregulation. Cdo1, Cysteine dioxygenase type 1; BMP4, Bone morphogenetic protein 4; MIF, Macrophage migration inhibitory factor; JNK, c-Jun N-terminal kinase; PI3K, Phosphatidylinositol-3 kinase; PPARα, Peroxisome proliferator-activated receptor alpha; PPARγ, Peroxisome proliferator-activated receptor gamma; AKT, Protein Kinase B; SIRT1, Sirtuin1; FOXO, Forkhead box O; Epac1, Exchange protein activated directly by cAMP 1.

## Data Availability

No new data were created or analyzed in this study. Data sharing is not applicable to this article.

## Supplementary Materials

The following supporting information can be downloaded at: https://www.mdpi.com/article/10.3390/metabo16040269/s1, Table S1: Comparison of traditional vs. molecular indicators for exercise prescription.

## Abbreviations

T2DM	Type 2 diabetes mellitus
cAMP	Cyclic adenosine monophosphate
PI3K-AKT	Phosphatidylinositol 3-kinase–protein kinase B
FOXO	Forkhead box O
MAPK	Mitogen-activated protein kinase
NAFLD	Non-alcoholic fatty liver disease
PKA	Protein kinase A
CREB	cAMP response element-binding protein
MRP4	Multidrug resistance-associated protein 4
CRTC2	CREB-regulated transcription coactivator 2
AMPK	AMP-activated protein kinase
PPARα	Peroxisome proliferator-activated receptor alpha
SREBP1	Sterol regulatory element-binding protein 1
IRS2	Insulin receptor substrate 2
SIRT1	Sirtuin1
JNK	c-Jun N-terminal kinase
ERK	Extracellular signal-regulated kinase
MAP4K4	Mitogen-activated protein kinase 4
GADD45	Growth arrest-and DNA damage-inducible protein 45
PPARγ	Peroxisome proliferator-activated receptor gamma
Cdo1	Cysteine dioxygenase type 1
CaMKK2	Ca2+/calmodulin-dependent protein kinase kinase 2
Epac1	Exchange protein activated directly by cAMP 1
HIIT	High-intensity interval training
MIF	Macrophage migration inhibitory factor
MKK4	Mitogen-activated protein kinase kinase 4
BMP4	Bone morphogenetic protein 4
GO	Gene Ontology
KEGG	Kyoto Encyclopedia of Genes and Genomes
