# Supplementary material for: Precision Exercise in Type 2 Diabetes Mellitus: Targeting Signaling Networks for Lipid Homeostasis
Source: Metabolites. 2026 Apr 16;16(4):269. doi: 10.3390/metabo16040269 (PMC13117746; doi:10.3390/metabo16040269)
Supplement: Supplementary file 1 [file metabolites-16-00269-s001.zip › metabolites-4239107-supplementary.pdf]

Supplementary Table S1. Comparison of traditional vs. molecular indicators for exercise prescription

	**Traditional indicators**	**Proposed molecular biomarkers (Exercise effector factors)**
**Examples**	Blood glucose, heart rate (HR), maximal oxygen uptake (VO_2max), rating of perceived exertion (RPE)	Cdo1, Epac1, PPARγ, PPARα, SIRT1, Spexin, BMP4, MIF
**Measured in**	Blood (glucose), real-time monitoring (HR, RPE), cardiopulmonary exercise test (VO_2max)	Blood (circulating levels) or tissue biopsy (e.g., skeletal muscle, liver)
**Reflects**	Immediate exercise intensity, systemic metabolic state, cardiovascular response	Signaling pathway-specific molecular adaptation (e.g., cAMP-Cdo1 axis, SIRT1-FOXO activity)
**Time window**	Real-time (during/immediately after exercise)	Delayed (days to weeks of exercise training)
**Clinical utility**	Cost-effective and easy to measure;<br>Ensures exercise safety (e.g., prevents overexertion);<br>Monitors acute responses	Reveals tissue-specific improvement in lipid homeostasis;<br>Could guide long-term exercise prescription adjustment;<br>Hypothesis-generating for personalized medicine
**Current status**	Standard care in clinical exercise prescription	Preclinical; requires validation in human trials
**Limitations**	Does not capture whether dyslipidemia is truly improved at the molecular level	Invasive (biopsy) or requires validated assays; not suitable for real-time monitoring
